# Exposure to Workplace Bullying, Distress, and Insomnia: The Moderating Role of the miR-146a Genotype

**DOI:** 10.3389/fpsyg.2019.01204

**Published:** 2019-05-24

**Authors:** Dhaksshaginy Rajalingam, Daniel Pitz Jacobsen, Morten Birkeland Nielsen, Ståle Valvatne Einarsen, Johannes Gjerstad

**Affiliations:** ^1^Department of Psychosocial Science, University of Bergen, Bergen, Norway; ^2^National Institute of Occupational Health, Oslo, Norway

**Keywords:** bullying, distress, insomnia, genotype, miR-146a, rs2910164

## Abstract

Several lines of evidence show that systematic exposure to negative social acts at the workplace i.e., workplace bullying, results in symptoms of depression and anxiety among those targeted. However, little is known about the association between bullying, inflammatory genes and sleep problems. In the present study, we examined the indirect association between exposure to negative social acts and sleep through distress, as moderated by the miR-146a genotype. The study was based on a nationally representative survey of 1179 Norwegian employees drawn from the Norwegian Central Employee Register by Statistics Norway. Exposure to workplace bullying was measured with the 9-item version of Negative Acts Questionnaire – Revised (NAQ-R) inventory. Seventeen items from Hopkins Symptom Checklist (HSCL-25) was used to measure distress. Insomnia was assessed with three items reflecting problems with sleep onset, maintenance of sleep and early morning awakening. Genotyping with regard to miR-146a rs2910164, previously linked to inflammatory processes, was carried out using Taqman assay. The data revealed that individuals systematically exposed to negative social acts at the workplace reported higher levels of sleep problems than non-exposed individuals. Moreover, the relationship between distress induced by exposure to negative social acts and insomnia was significantly stronger for individuals with the miR-146a GG genotype. Thus, the miR-146a genotype moderated the association between distress and insomnia among individuals exposed to negative social acts. The present report support the hypothesis that inflammation could play a role in stress-induced insomnia among individuals exposed to workplace bullying.

## Introduction

Exposure to bullying at the workplace, be it from one’s peers or one’s superiors, is a prevalent social stressor with severe consequences in those targeted (Nielsen and Einarsen, 2012). Representing a systematic form of exposure to workplace mistreatment, the term “bullying” refers to a situation in which a person repeatedly is subjected to negative social acts in a situation, where the target is unable to defend him/herself (Einarsen and Skogstad, 1996; Gredler, 2003). Bullying is not an either or phenomenon, but rather a gradually escalating process ranging from single acts of incivility to systematic exposure to aggression and social exclusion at work. To this date, most research on outcomes of bullying has focused on mental distress and has established bullying as a significant predictor of depression and anxiety in targets (Hansen et al., 2011). The empirical evidence for an association between bullying and sleep is; however, more scarce. Yet, from a bio-physiological perspective, it is theoretically plausible that systematic exposure to bullying-related stress at work also affects sleep via elevated levels of distress. For example, exposure to negative social acts may induce mental distress caused by cognitive rumination and persistent central nervous system (CNS) activation – which in turn could be associated with sleep problems (Akerstedt, 2006; Fortunato and Harsh, 2006; Han et al., 2012).

Exposure to negative social acts is a strong stressor that may affect both the hypothalamus in the brain stem and the autonomous nervous system (ANS). Thus, an alternative explanation for an association between exposure to negative social acts and sleep is that the exposure may lead to a disturbed balance between the parasympathetic and sympathetic branch of the ANS, i.e., reduced acetylcholine (Ach) and more norepinephrine (NE) release close to the ANS target organs (Mineur et al., 2013; Won and Kim, 2016). Moreover, exposure to systematic negative social acts, through the sympatho-adreno-medullary connections, increase the release of circulating catecholamines. Exposure to negative social acts also activates the hypothalamic-pituitary-adrenal (HPA) axis, which promote release of corticotrophin releasing hormone (CRH), adrenocorticotrophic hormone (ACTH) and cortisol (Akerstedt, 2006).

Interestingly, reduced parasympathetic or increased sympathetic activity following exposure to negative social acts may promote inflammatory processes in circulating immune cells through the influence on the spleen and other lymphoid tissues. Such stress-induced autonomic influence on lymphoid tissues, may be associated with low-grade systemic inflammation, which in turn could be linked to sleep problems (Motivala, 2011). In addition, in the initial stage of sleep, the level of ACTH and cortisol is reduced. This suppresses the activity of HPA axis and induces sleep. In the later stage, before awakening, HPA axis activity increases. Accordingly, the rise of ACTH in the morning controls the end of sleep (Akerstedt, 2006). Therefore, increased HPA axis activity due to distress, will most likely also cause insomnia.

Stress-induced changes in the immune system involves many innate immune cells i.e., lymphoid and myeloid cells, which release circulating cytokines (Chrousos, 1995; Turnbull and Rivier, 1995). Over time this could be a threat to homeostasis of the immune system (Turnbull and Rivier, 1995) and is therefore, maladaptive (Wohleb et al., 2015). Thus, chronic stress, including exposure to bullying, may be associated with many negative physiological and immunological changes (Chrousos, 1995; Wohleb et al., 2015). Increasing evidence support the idea that microRNAs (miRs), RNA molecules of ∼ 22 nucleotides in length, play key roles in these immunological processes (McDonald and Ajit, 2015). The miRs bind to messenger RNA (mRNA) and inhibit translation of mRNA to proteins by binding to complementary sequences in the 3′ untranslated region of a specific mRNA target. Alternatively, miR-binding to the complementary sequence can result in degradation of the mRNA.

A crucial protein complex controlled by the ANS efferents to the spleen, which also influences systemic inflammatory processes, may be the NF-*κ*B (nuclear factor kappa-light-chain-enhancer of activated B cells). Interestingly, activation of the NF-*κ*B pathway in circulating monocytes or other immune cells results in up-regulation of many inflammatory cytokines, but also miR-146a – which in turn targets upstream proteins and further modulate the inflammatory response (Saba et al., 2014). Therefore, the gene encoding miR-146a (Baltimore et al., 2008; Saba et al., 2014), has been implicated to play a central role in regulating the innate immune response (Saba et al., 2014; Lee et al., 2016). Given that low-grade systemic inflammation promotes insomnia (Motivala, 2011), the miR-146a rs2910164 G allele that supports inflammatory processes (Shen et al., 2008), may also affect sleep.

**FIGURE 1:**
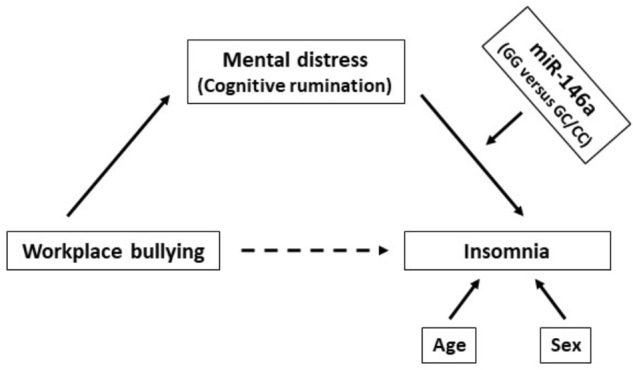
A graphic illustration of the proposed relationship between workplace bullying, distress and insomnia moderated by the miR-146a genotype (adjusted for the covariates age and sex).

Several lines of evidence show that miR-146a may be a dominant, negative regulator of the innate immune response (Saba et al., 2014; Lee et al., 2016). For example, miR-146a can target mRNA of proteins in the NF-*κ*B pathway, which in turn may regulate the expression of a cluster of cytokines including IL-1 and TNFα. Interestingly, it has been proposed that miR-146a may influence on Toll-like receptor and cytokine signaling in monocytes through a negative feedback loop involving down-regulation of IL-1 receptor-associated kinase 1 and TNF receptor-associated factor 6 protein levels (Taganov et al., 2006). Moreover, nitric oxide synthase 1 (NOS1), an important retrograde signaling molecule in the CNS that also affects peripheral inflammatory processes, is reported to be a target of miR-146a (Zhang et al., 2018). Therefore, based on the link between stress-induced inflammation and sleep – and the fact that miR-146a may control both IL-1, TNFα and NOS1 – we hypothesized that the relationship between distress and insomnia may be amplified by the miR-146a rs2910164 GG genotype. A graphical illustration of the proposed relationship investigated in the present study is shown in Figure 1.

## Materials and Methods

### Design and Sample

This study is based on a probability survey of the Norwegian workforce. A random sample of 5000 employees was drawn from The Norwegian Central Employee Register by Statistics Norway. The Norwegian Central Employee Register is the official register of all Norwegian employees, as reported by employers. Sampling criteria were adults from 18 to 60 years of age employed in a Norwegian enterprise. Questionnaires were distributed through the Norwegian Postal Service during spring 2015. Altogether 1608 persons returned the questionnaire (32%) and all respondents provided usable responses. Subjects who gave consent were also sent saliva collection kits. Among these, 1204 returned the saliva sample kit. The analyses were; however, performed with 1179 subjects due to missing data. The survey was approved by the Regional Committee for Medical Research Ethics for Eastern Norway. Responses were treated anonymously, and informed consent was given by the respondents.

Mean age was 45.19 (SD = 10.04) years with a range from 21 to 61 years. The sample consisted of slightly more women (52.1%) than men (47.8%). In total, 54.9% were married, 24.5% were common-law partners, 13.8% were unmarried, and 6.8% were widowed, separated, or divorced. Altogether 8.4% had less than 11 years of education, 30.8% had between 11 and 13 years, 32.3% had between 14 and 17 years, and 28.5% had 18 or more years. A total of 89.6% were in a full-time employment, 6.6% were in part-time employment, 3.5% were on a sick leave or occupational rehabilitation, and 0.3% were disabled pensioners or retired. Moreover, 36% had a leadership position with personnel responsibilities. Comparisons of sample characteristics with available data from Statistics Norway suggested that the sample distribution was somewhat skewed compared to the overall working population with regard to gender (53% men in population), educational level (less than 11 years of education: 17%; between 11 and 13 years: 42%; more than 14 years: 41% in population), and age mean of 41.8 years in population.

### Instruments

Exposure to negative social acts at the workplace was measured with the 9-item version of the Negative Acts Questionnaire – Revised (NAQ-R) inventory (Einarsen et al., 2009). NAQ-R describes negative and unwanted behaviors that may be perceived as bullying if occurring on a regular basis. All items are formulated in behavioral terms and hence, focus on the mere exposure to inappropriate behaviors while at work with no references to the term bullying (Einarsen and Nielsen, 2015). The NAQ-R contains items referring to both direct (e.g., openly attacking the victim) and indirect (e.g., social isolation, slander) behaviors (Einarsen et al., 2009). The items do also distinguish between personal and work related forms of bullying (Einarsen et al., 2009). Example items are “Being ignored or excluded,” “Repeated reminders of your errors or mistakes,” and “Someone withholding information which affects your performance.” The respondents were asked to indicate how often they had been exposed to each specific item in questionnaire at their present worksite during the last 6 months. Response categories ranged from 1 to 5 (‘never,’ ‘now and then,’ ‘monthly,’ ‘weekly,’ and ‘daily’). This nine item version of the NAQ-R had a Cronbach’s alpha of 0.86 in this study.

Seventeen items from Hopkins Symptom Checklist (HSCL-25) reflecting typical symptoms of anxiety and depression measured *symptoms of psychological distress* during the last week. The HSCL is a valid and reliable (Rickels et al., 1976) self-administered instrument measuring mental distress (anxiety, depression, and psychosomatic complaints) in population surveys (Derogatis et al., 1974). Earlier comparisons show that shorter versions perform as well as the more extensive versions of the inventory (Strand et al., 2003). Responses were given on a four-point scale, ranging from “1 = not at all” to “4 = extremely.” Example items are “Feeling no interest in things” and “Feeling hopeless about the future.” Cronbach’s alpha for this scale was 0.87 in the current study.

Insomnia was assessed with three items reflecting problems with sleep onset, maintenance of sleep and early morning awakening. Response categories ranged from 1 to 4 (‘not bothered,’ ‘a little bothered,’ ‘considerably bothered,’ ‘seriously bothered’). These symptoms are core nocturnal characteristics of insomnia, in line with modern nosology (American Psychiatric Association, 2013; American Academy of Sleep Medicine, 2014). A composite insomnia score was calculated by adding the score of the three items and dividing the sum by three. The Cronbach alpha for the insomnia scale was 0.81 in the present study.

### Genotyping

As previously described (Jacobsen et al., 2018), genomic DNA was extracted from saliva using an OrageneRNA sample collection kit (DNA Genotech Inc., Kanata, Ontario, Canada). Single nucleotide polymorphism (SNP) genotyping was carried out using predesigned TaqMan SNP genotyping assays (Applied Biosystems, Foster City, CA, United States). Approximately 10 ng genomic DNA was amplified in a 5 μl reaction mixture in a 384-well plate containing 1x TaqMan genotyping master mix (Applied Biosystems) and 1x assay mix, the latter containing the respective primers and probes. The probes were labeled with the reporter dye FAM or VIC to distinguish between the two alleles. After initial denaturation and enzyme activation at 95°C for 10 min, the reaction mixture was subjected to 40 cycles of 95°C for 15 s and 60°C for 1 min on an ABI 7900HT sequence detection system. Negative controls were included in every run. Genotypes were determined using the SDS 2.2 software (Applied Biosystems, Foster City, CA, United States). Approximately 10% of the samples were re-genotyped and the concordance rate was 100%.

### Statistical Analysis

Exposure to negative social acts was calculated using the mean-score of the 9 items in the NAQ-R inventory. Since we in our sample had 759 GG subjects, 401 GC subjects, but only 45 CC subjects, the miR-146a genotype was included as a dichotomous variable, GG versus GC/CC. To investigate the hypotheses about main and moderating effects, we conducted a moderated mediation regression analysis using a modeling tool, SPSS; PROCESS v3.1, to test for linear associations between exposure to negative social acts and insomnia, as well as the interactive effects of negative social acts and miR-146a genotype (GG versus GC/CC) with regard to insomnia. Deviation from the Hardy–Weinberg equilibrium was tested by the Chi-squared test (Chi^2^ = 0.7936).

As discussed in the introduction of this manuscript, there are theoretical reasons for expecting that the impact of workplace bullying on insomnia is mediated by psychological distress, and that the magnitude of this relationship is conditioned by miR-146a rs2910164 genotype. Specifically, we hypothesized that bullying is expected to increase levels of distress and the increased levels of distress is further expected to lead to insomnia among employees with the GG genotype. To empirically test this theoretical relationship we analyzed a moderated mediation model.

A mediation model is one that seeks to identify and explain the mechanism or process that underlies an observed relationship between an independent variable and a dependent variable via the inclusion of a third variable (i.e., mediator variable), in our case between bullying and insomnia. According to Baron and Kenny (1986), there is evidence that a variable mediates the relationship between a predictor variable and an outcome variable when each of the following conditions have been met: (a) there is a significant relationship between a predictor (e.g., exposure to workplace bullying) and an outcome (e.g., insomnia), (b) there is a significant relationship between a predictor and a proposed mediator variable (e.g., psychological distress), (c) there is a significant relationship between a proposed mediator and an outcome (with the predictor controlled), and (d) the strength of the relationship between a predictor and an outcome decreases significantly when a proposed mediator is controlled (Frazier et al., 2004). A full mediation is supported when a predictor variable is no longer significantly associated with the outcome after adjusting for the mediating variable. A moderated mediation model is supported if the magnitude of the indirect association between the predictor and outcome variable through the mediator is conditionally dependent upon the values of the moderator variable (e.g., in our case, miR-146a rs2910164).

The hypothesized moderated mediation model was tested in full by means of the PROCESS macro (model 14) developed for SPSS. PROCESS uses an ordinary least squares (OLS) or logistic regression-based path analytical framework for estimating indirect effects in both un-moderated and moderated mediation models with a single or multiple mediators and moderators (Hayes, 2013). Bootstrap methods are implemented in PROCESS for inference about indirect effects in both unmoderated as well as moderated mediation models. Bootstrapping is a statistical procedure that allows for the calculations of effect sizes even when you do not know the underlying distribution. The analysis was adjusted for age and sex, as covariates. A significant interaction term and a significant increase in explained variance (R^2^) were considered as indicative of an interaction effect.

**Table 1:** Characteristics of the subjects grouped by the miR-146a genotype rs2910164; GG versus GC/CC.

	Range	GG				GC/CC				Sum	*T*-test
		N	%	Mean	SEM	N	%	Mean	SEM		
Subjects		758	62.9			446	37			1204	
Insomnia	1 to 4			1.71	0.027			1.64	0.315		1.50
NAQ	1 to 5			1.18	0.011			1.21	0.017		−1.77
Distress	1 to 4			1.36	0.013			1.37	0.018		–0.51
Age				46	0.813			44.5	0.465		
Male		378	49.8			200	44.8				
Female		380	50.1			246	55.2				
Education											
Secondary school or less		20	2.6			6	1.3				
High school		277	36.5			169	37.9				
University ≤ 4 years		237	31.3			149	33.4				
University ≥ 4 years		222	29.3			119	26.7				

*NAQ, Negative Acts Questionnaire; SEM, Standard error of the mean.*

As the scores on the NAQ-R (skewness: 4.18, kurtosis: 26.85) were non-normally distributed, all analyses were conducted using bootstrapping (5000 resamples). The bootstrap method has the advantage that it does not need to meet the assumptions of normality, equal variances and homoscedasticity that are required in ordinary regression analyses. Multicollinearity was not an issue in the current study (VIF = 1.01). The level of significance was set to *p* < 0.05.

## Results

The present data showed that 55% of the individuals included in our probability sample reported exposure to at least one negative act; NAQ > 1 at the workplace during the last 6 months. Mean negative acts scores were similar for men and women; NAQ = 1.18. The mean insomnia scores for men and women were 1.64 and 1.72, respectively. ANOVA analyses with age and gender as covariates showed no significant differences between genotypes with regard to scores on NAQ, insomnia, and distress.

The characteristics of the subjects are presented in Table 1. As expected, genotyping demonstrated that the majority, i.e., 63%, of the subjects had the ordinary variant GG, whereas the rest, i.e., 37%, carried the rare variant GC/CC. No deviation from the Hardy–Weinberg equilibrium was observed.

**Table 2:** Regression analysis SPSS PROCESS model 14 with the miR-146a genotype rs2910164; GG versus GC/CC (bootstrapping with 5000 samples).

	B	SE	*P*-value	95% CI
*Distress*				
NAQ	0.3668	0.287	0.0000	0. 3104 to 0.4232
Age	−0.0006	0.0005	0.2425	−0. 0016 to 0.0004
Sex	0.0954	0.0194	0.0000	0. 0573 to 0.1335
*Insomnia*				
NAQ	0.3188	0.0600	0.0000	0. 2011 to 0.4366
Age	0.0043	0.0010	0.0000	0. 0023 to 0.0063
Sex	0.0182	0.0384	0.6356	−0.0571 to 0.0935
Distress	0.6752	0.0571	0.0000	0. 5632 to 0.7872
miR-146a GG^∗^ vs. GC/CC	−0.0813	0.0394	0.0391	−0.1585 to −0.0041
Distress x miR146a GG^∗^ vs. GC/CC	−0.4337	0.1080	0.0001	−0.6457 to −0.2218

*^∗^reference group. The analysis were adjusted for the covariates age and sex. SE, standard error; CI, confidence interval.*

**FIGURE 2:**
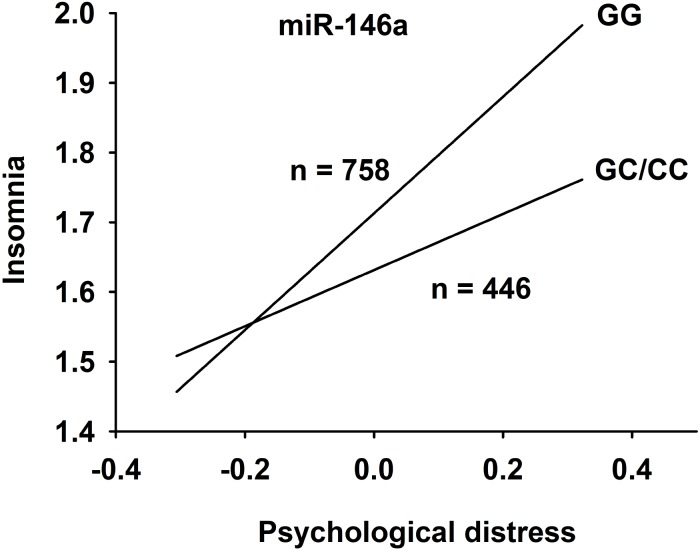
The relationship between psychological distress and insomnia after correction for age and sex. Subjects were divided into groups based on miR-146a genotype rs2910164; GG versus GC/CC.

The data from the moderated mediation analysis is presented in Table 2. Analyses of direct associations between the predictor and outcome variables in the hypothesized model showed that, after adjusting for age and gender, exposure to negative acts, i.e., elevated NAQ score, was associated with insomnia (*b* = 0.55; *p* < 0.001). Negative acts and the control variables explained eight percent of the variance in insomnia. Exposure to negative acts was also significantly associated with distress (*b* = 0.37; *p* < 0.001), and explained 13.9% of the variance in distress. When including the mediator (distress) and the moderator (miR-146a GG versus GC/CC) variables in the analyses of the association between exposure to negative acts and insomnia, the findings showed that exposure to negative acts had an indirect (mediated) association with insomnia through distress for both genotypes (see Table 2). However, a significant interaction term showed that this indirect effect was stronger among individuals with the GG genotype (*B* = 0.31; 95% CI: 0.23–0.41) than among individuals with the GC/CC genotype (*B* = 0.16; 95% CI: 0.08–0.23). Hence, the present data revealed that there was an indirect relationship between exposure to negative acts and insomnia through distress and that the magnitude of this indirect relationship was stronger for individuals with GG than for individuals with GC/CC (Figure 2 and Supplementary Figure S1). When including the mediator and the interaction term in the model, the variables explained 19% of the variance in insomnia.

## Discussion

In the present study, we demonstrated that individuals systematically exposed to negative social acts at the workplace report higher levels of sleep problems than non-exposed individuals. Our data also demonstrated that this association may be strengthened among individuals having the miR-146a rs2910164 GG genotype. Since previous observations show that miR-146a may be upregulated in, but also is a regulator of inflammatory processes, the present data suggest that inflammation could play a role in stress-induced insomnia among individuals exposed to negative social acts.

Over the last 20 years, there has been an evolving understanding of the bidirectional communication between the CNS and the immune system (Krueger and Majde, 2003), which also provides the network for sleep regulatory circuits in the brain (Davis and Krueger, 2012). The important roles of cytokines as signaling molecules in this communication and their ability to bypass the blood-brain-barrier has also been recognized. Several lines of evidence show that cytokines, i.e., IL-1 and TNFα through their influence on neuronal signaling regulates sleep and enhance non-rapid eye movements (Krueger and Majde, 2003; Del Gallo et al., 2014). Studies also show that variation in plasma levels of IL-1 and TNFα are associated with sleep quality in patients with chronic inflammation (Krueger et al., 2011). The correlation between cytokine levels, sleep and pathology support the hypothesis that a low-grade systemic inflammation induced by chronic stress, in our case social stress, could cause changes in circulating cytokine levels, which influence on sleep circuits in the brain (Olini et al., 2017).

Previous data show that miR-146a targets mRNA of proteins in the NF-*κ*B pathway in circulating monocytes and that miR-146a therefore may attenuate the innate immune response (Saba et al., 2014). A study performed by Shen and colleagues (Shen et al., 2008) shows that the rs2910164 G allele results in reduced levels of expression of the anti-inflammatory miR-146a in MCF-7 cells, a breast cancer cell-line. This shows that the G allele could promote low-grade systemic inflammation and sleep problems. However, other studies suggest that the G allele also may have the opposite effect due to the stability of the pre-miR (Jazdzewski et al., 2008; Xu et al., 2008). Apparently, the miR-146a G > C polymorphism may have different effects in different tissues (Park et al., 2016).

Recently, the nitric oxide synthase 1 (NOS1) has been reported to be a direct target of miR-146a (Zhang et al., 2018), meaning that the NOS1 expression would be affected by the miR-146a G > C polymorphism (Luan et al., 2016). NOS1 is an enzyme, responsible for the production of nitric oxide (NO) – an important pro-inflammatory molecule and a retrograde signaling messenger in the CNS. Previous data show that NOS1 and the nitric oxide pathway is directly linked to the HPA axis and the regulation of glucocorticoids (Chen et al., 2015). In addition, NOS1 may be involved in psychological distress (Luciano et al., 2012), suggesting that miR-146a polymorphism could have an effect on depression and anxiety. It is tempting to speculate that miR-146a could influence on the neuronal processes underlying psychological distress, which in turn affect immunity and sleep. This demonstrates the capability of miRs in regulating neural circuits important for stress-induced insomnia and other health complaints.

Being based on cross-sectional data, however, the present study has its limitations. Moreover, the study design causes problems explaining causal relationships. In addition, as the measurement instruments for negative social acts and insomnia were self-report measures, the study could be influenced by bias such as set tendencies and social desirability. Also, the overall response rate for the questionnaire survey was only 32%, and <20% of the invited participants returned their saliva samples. Thus, we cannot be certain that the final sample is representative for the overall population. Nevertheless, as response rate and representatively seems to have limited impact on the internal validity (Schalm and Kelloway, 2001), the response rate may not really be a problem with regard to our findings.

In summary, the present data suggest that exposure to bullying-related negative social acts at the workplace may lead to increased risk of sleep problems through elevated levels of mental distress. Moreover, our data show that the link between distress and insomnia may be moderated by the miR-146a genotype, i.e., the rs2910164 G > C polymorphism within the precursor sequence of miR-146a. Hence, the present study indicate that the effect of systematic exposure to negative social acts at work on insomnia among those that are targeted is strengthened in individuals with the miR-146a genotype GG. Thus, it is important that such biological factors are taken into account when future intervention studies are designed. In particular, the interaction between exposure to negative social acts, genetics and insomnia should be acknowledged. Such knowledge could be of vital importance when treating and rehabilitating patients who have suffered mental health problems after exposure to workplace bullying and other forms of social stress and mistreatment while at work. We conclude that the association between distress and insomnia among individuals exposed to negative social acts is moderated by genetic variability in the gene encoding miR-146a.

## Ethics Statement

This study was carried out in accordance with the recommendations of “Regional Committee for Medical Research Ethics for Eastern Norway” with written informed consent from all subjects. All subjects gave written informed consent in accordance with the Declaration of Helsinki. The protocol was also approved by the “Regional Committee for Medical Research Ethics for Eastern Norway.”

## Author Contributions

DR, DJ, MN, SE, and JG designed the research. DR, DJ, and JG performed the research. DR and MN analyzed the data. DR and JG wrote the manuscript. All authors have commented on, read and approved the final manuscript.

## Conflict of Interest Statement

The authors declare that the research was conducted in the absence of any commercial or financial relationships that could be construed as a potential conflict of interest.

## Supplementary Material

FIGURE S1 The effect of miR-146a genotype on insomnia in low, median, and high distress individuals.
