# Supplementary figures and images for: Exposure to Workplace Bullying, Distress, and Insomnia: The Moderating Role of the miR-146a Genotype
Source: Front Psychol. 2019 May 24;10:1204. doi: 10.3389/fpsyg.2019.01204 (PMC6542980; doi:10.3389/fpsyg.2019.01204)

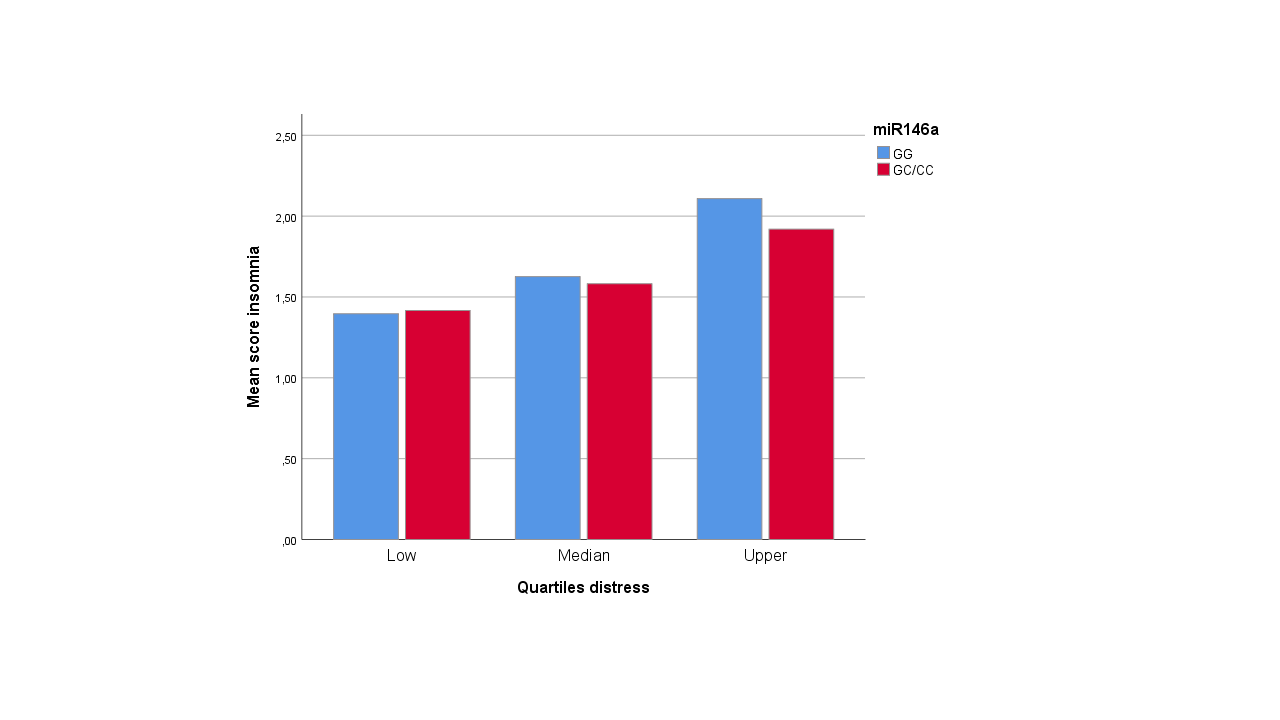

Supplement: FIGURE S1 — The effect of miR-146a genotype on insomnia in low, median, and high distress individuals. [file Image_1.TIF]
